# S1PR1 regulates the switch of two angiogenic modes by VE-cadherin phosphorylation in breast cancer

**DOI:** 10.1038/s41419-019-1411-x

**Published:** 2019-02-27

**Authors:** Shuang Liu, Chunsheng Ni, Danfang Zhang, Huizhi Sun, Xueyi Dong, Na Che, Xiaohui Liang, Chen Chen, Fang Liu, Jingru Bai, Xian Lin, Xiulan Zhao, Baocun Sun

**Affiliations:** 1 Department of Pathology, Tianjin Medical University, Tianjin, China; 2 Department of Pathology, General Hospital of Tianjin Medical University, Tianjin, China; 3 Department of Pathology, Cancer Hospital of Tianjin Medical University, Tianjin, China

## Abstract

Angiogenesis in solid tumors is divided into two modes: endothelium-dependent vessel (EDV) and vasculogenic mimicry (VM). Sphingosine-1-phosphate receptor 1 (S1PR1) plays a vital role on EDV in a variety of human tumors. However, the relationship between S1PR1 and VM is not clear. The aim of this study is to investigate S1PR1 on the regulation of EDV and mimicry formation in breast cancer. Here we show that S1PR1 phosphorylates the complex of VE-cadherin to regulate the switch of EDV and mimicry formation. Suppression of S1PR1 impairs EDV, but contributes to the generation of VM, invasion, and metastasis in vivo and vitro. By inhibiting RhoA activation, the S1PR1/VE-cadherin signaling is blocked. S1PR1 controls VE-cadherin expression and EDV via RhoA activation. Moreover, the low expression of S1PR1 correlates with VM and poor prognosis in breast cancer patient. The results show that S1PR1 regulated RhoA activation to accelerate VE-cadherin phosphorylation (Y731), leading to increased EDV and reduced VM in breast cancer. S1PR1 may provide a new thinking direction for antiangiogenic therapy for patients with breast cancer.

## Highlights

1. The deficiency of S1PR1 contributes to the generation of VM.
2. S1PR1 could induce the switch between EDV and VM formation in human breast cancer.
3. S1PR1 might provide a new direction for antiangiogenic therapy for patients with breast cancer.

## Introduction

The incidence and mortality rates of breast carcinoma have steadily increased in many countries. Among females, breast cancer is the most commonly diagnosed cancer and is the leading cause of cancer death^1^. Angiogenesis is an indispensable aspect of tumor growth and metastasis in breast cancer and other solid tumors^2^. Tumor angiogenesis has been proven to be functionally inferior and immature. Vasculogenic mimicry (VM) is a vessel-like network that lacks endothelial cells in which the tumor cells coexpress endothelial and tumor markers^3^. VM is strongly involved in a variety of malignant human tumors, including breast cancer^4–8^. VM contributes to a poor prognosis, tumor metastasis, poor 5-year overall survival, and increased patient mortality^9^. Some signaling molecules regulate endothelium-dependent blood vessel (EDV), including vascular endothelial growth factor (VEGF) and platelet-derived growth factor (PDGF)^2^. The mechanisms and signaling pathways for VM formation include vascular endothelial-cadherin (VE-cadherin)^10,11^, epithelial cell kinase (EphA2)^12^, phosphoinositide 3-kinase (PI3K), and focal adhesion kinase (FAK)^13^. In the process of tumor development, the two angiogenesis modes can be converted to each other. The intermediate form of transition is called a mosaic blood vessel. The mechanism by which factors participate in the transition between the two angiogenesis modes is not completely understood. Because of the complexity, single antiangiogenic therapy is unsatisfactory^14^.

Sphingosine-1-phosphate (S1P) is a bioactive signaling lipid generated by sphingosine kinase (Sphk)^15,16^. S1P is a regulator of vascular development and function, including vascular maturation^17,18^. S1P receptor 1 (S1PR1) is a G-protein-coupled receptor for S1P and a biologically active metabolite of sphingolipid^19^. When S1PR1 regulates cell-to-cell interactions, Rho (a small guanine nucleotide binding protein) is usually its downstream binding protein^20^. Several studies have shown that S1PR1 has a necessary role in several tumors^16,21–24^. Therefore, to inhibit angiogenesis in tumor cells, a Sphk inhibitor was used to inhibit S1P synthesis in tumor cells and decrease tumor viability and growth^14,25^. However, previous results are contradictory, possibly because the dual angiogenesis patterns prevent S1PR1-related signals from blocking EDV but cause tumor cells to produce self-sufficient blood supply patterns (VM).

In our study, we demonstrate that S1PR1 promotes EDV, and that S1PR1 deficiency contributes to the generation of VM. Knockdown of S1PR1 in breast cancer cells increased the amount of VM. Tube formation by human umbilical vein endothelial cells (HUVECs) was increased after treatment with conditioned medium (CM) from the S1PR1 overexpression group. S1PR1 promotes the separation of VE-cadherin from β-catenin by increasing VE-cadherin phosphorylation. This process was mediated through RhoA activation. Tumor cells in the low S1PR1 group obtained nutrients through VM, and tumor growth was accelerated in animal models.

## Materials and methods

### Clinical samples

One hundred breast cancer specimens were obtained from the General Hospital of Tianjin Medical University (Tianjin, China). These specimens were collected from patients between 1997 and 2005. The diagnosis of breast cancer in these samples was verified by two or more pathologists. Detailed pathological and clinical data were collected for all samples. The use of these tissue samples was approved by the Ethics Committee of Tianjin Medical University.

### Immunohistochemistry

The tissues were deparaffinized in xylene and rehydrated in graded alcohols. First, 3% H_2_O_2_ was used to block endogenous peroxidase, followed by antigen retrieval. Tissue sections were blocked in 10% goat serum (Zhongshan Chemical Co., Beijing, China) and incubated consecutively with primary antibodies and a secondary antibody. The results were scored on a scale of 0–3 based on the percentage of tumor cells stained as follows: 0 (negative), 1 (weak, ≤ 25%), 2 (medium, 25%–50%), and 3 (high, > 50%). The samples were further divided into negative (score < 2) and positive (score ≥ 3) score categories. For patients with clear immunohistochemistry (IHC) staining and survival follow-up data, we analyzed the correlation between S1PR1 and survival information, the numbers of VM events and the EDV, and other related indicators.

### Cell culture

The human breast cancer cell lines HS-578T, MDA-MB-231, MCF-7, T-47D, and BT-474 and HUVECs were obtained from the ATCC in 2012 and all cell lines underwent verification in January 2014. The cells were cultured in Dulbecco’s modified Eagle’s medium (Neuronbc) containing 10% fetal bovine serum (FBS; Pro) and 1% antibiotics (penicillin and streptomycin). All cells were cultured at 37 °C with 5% CO_2_.

### Cell transfection and preparation of CM

We used HEK293T cells for lentivirus production, purification, and infection according to the manufacturer’s instructions (Lenti-PacTM HIV Expression Kit, Genecopoeia). The plasmids were synthesized by Genecopoeia, including S1PR1 complementary cDNA (EX-Z2508-LV206), a negative control (EX-NEG-Lv201), S1PR1 small interfering RNAs (HSH004554-LVRU6GP), and a shControl (CSHCTR001-LVRU6GP). MDA-MB-231 cells were transfected with a S1PR1 expression plasmid or control vector plasmid, and MCF-7 cells were infected with a lentivirus containing shS1PR1 or shControl for 24 h. The transfected cells were grown to 70–80% confluency, washed three times with phosphate-buffered saline (PBS) and then incubated in fresh medium containing 10% FBS for 48 h. HUVECs in the coculture experiments needed CM. Transfected tumor cells were starved for 24 h in serum-free medium, which then was replaced with 5% FBS medium. CM was harvested, centrifuged at 1000 rpm for 10 min to remove cell debris, filtered through a 0.22-µm filter, and stored at 4 °C.

### Three-dimensional (3D) cell cultures

The bottom of a 96-well plates was tiled with Matrigel (BD, USA). The 96-well plate was irradiated and dried on an ultraclean bench and then placed in a 37 °C cell incubator for hydration. Tumor cells were suspended in culture medium and added to the 96-well plate and incubated for 24–36 h at 37 °C. An inverted microscope captured the number of VM tubes. Each condition was performed for at least three independent experiments.

### Cell proliferation assay

We resuspended 2000 cells in 96-well plates. The cells were monitored every 24 h for 5 days. For the detailed steps, please see the MTT product brochure (Key Gene catalog no.: KGA311/312). The results were measured using the Synergy 2 plate reader (Bio Tek).

### Western blot assay

Equal amounts of protein from cell lysates were separated by Sodium dodecylsulphate polyacrylamide gel electrophoresis (SDS–PAGE) and transferred to polyvinylidene fluoride membranes (Millipore, MA, USA), which were incubated with various antibodies (the details are provided in the Supplementary Materials). Glyceraldehyde-3-phosphate dehydrogenase was used as an internal loading control.

### In vitro migration and invasion assays

For the migration and invasion assays, 2 × 10^4^ cells were seeded in serum-free medium in the upper chambers of Transwells (Invitrogen) with or without Matrigel. Culture medium containing 10% FBS was added to the bottom chamber. Infected MDA-MB-231 cells were incubated at 37 °C and allowed to migrate for 24 h or invade through the Matrigel for 48 h. Infected MCF-7 cells were incubated for an additional 12 h. The cells on the upper membrane were removed with a cotton swab. Cells on the lower surface of the membrane were fixed with cold methanol and stained with 0.1% crystal violet for 40 min. Five Transwell fields were photographed using an inverted optical microscope (Nikon). Image analysis software (Image-Pro Plus 6.0; Media Cybernetics) was used to estimate the cell density.

### Immunofluorescence staining

The cells were tiled on coverslips, incubated at 37 °C overnight, permeabilized with 0.1% Triton X-100 and blocked with 5% FBS. Then, the cells were incubated with primary antibodies against S1PR1 (ab11424; Abcam; 1:50), VE-cadherin (ab33168; Abcam; 1:50), and β-catenin (ab32572; Abcam; 1:100). After incubation with fluorophore-conjugated secondary antibodies, the nuclei were counterstained with 4',6-diamidino-2-phenylindole(DAPI) (Sigma). Images were acquired by fluorescence microscopy (Nikon, Japan).

### Enzyme-linked immunosorbent assay (ELISA)

The human VEGF immunoassay kit from Abcam (Cambridge, MA, USA) was used to measure the VEGF concentrations in the tumor cell supernatants. Samples were prepared following the manufacturer’s protocol.

### G-LISA activation assays

The key step in the sample preparation process is to immediately place the dish on ice and keep it at low temperature throughout the process. The cells were quickly washed with ice-cold PBS, and the wash solution was carefully removed completely. The cells were shaken at 4 °C using the minimum volume of ice-cold lysis buffer (70 µl/8 cm^2^) required for efficient cell lysis. After 5 min, the lysate was centrifuged (10,600 *g*, 2 min, 4 °C) and kept on ice, and the protein concentration was determined by measurement. Specific steps refer to the manufacturer’s instructions for the Small G-protein Activation Assay (G-LISA) activation assay kits (Cytoskeleton).

### Animal studies

The experiment followed the Institutional Animal Care and Use Committee (IACUC) guidelines, and the ethical review was approved by the Tianjin Medical University IACUC committee. Forty-eight BALB/c-nu/nu mice were purchased from IACUC and divided into four groups by volume and weight. The established MDA-MB-231-S1PR1 expression and MCF-7-S1PR1-sh cell lines and the corresponding control group cell lines were injected into the nude mice. The cell lines were mixed with PBS and Matrigel (1:1) at a cell concentration of 8×10^6^/100 µl and injected into the mice. The volume of the transplanted tumor in the mice was measured, and a growth curve was drawn. The mouse tumors were fixed and analyzed by immunohistochemistry.

### Statistical analyses

Statistical analyses were performed with SPSS 22.0 (SPSS Inc., Chicago, IL, USA). All data were repeated independently in at least three experiments. Values are expressed as the mean ± SD (standard deviation). Student’s *t*-test was used to analyze differences between two groups. The chi-squared test was used to compare categorical variables. Differences in breast cancer specimens were compared by analysis of variance. *P*-values of <0.05 were considered to be statistically significant.

## Results

### Clinical significance of S1PR1 in tumor tissues from breast cancer patients

**Fig. 1:**
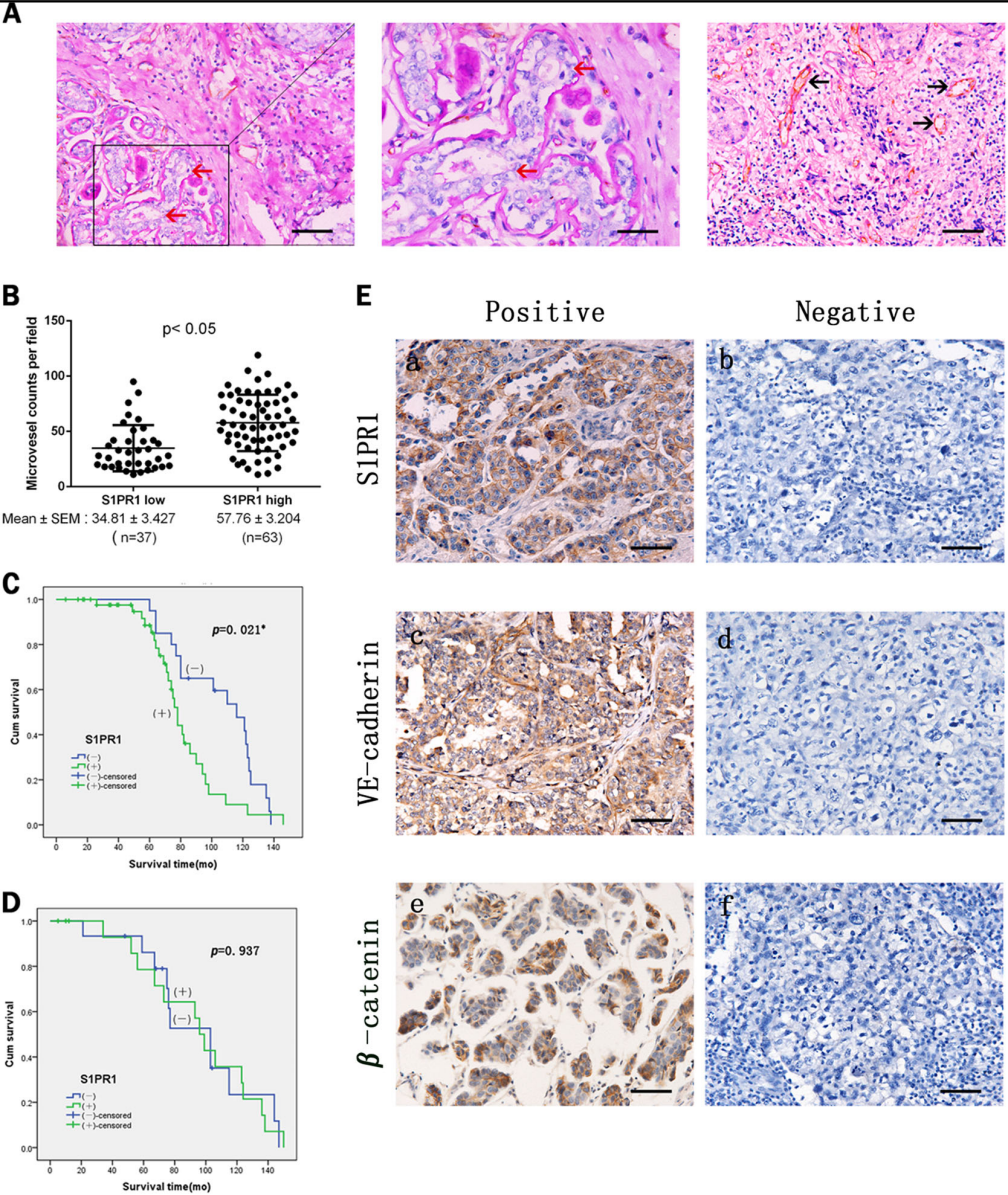
Sphingosine-1-phosphate receptor 1 (S1PR1) expression correlates with vasculogenic mimicry (VM) and endothelium-dependent vessel (EDV) in human breast cancer tissues. **a** CD31/PAS double staining shows the VM channels in human breast cancer specimens. The channels (red arrowhead) lined with tumor cells contained red blood cells and were CD31 negative and PAS positive ( × 200, bars 20 µm). The EDVs were CD31-positive (black arrowhead) ( × 200, bars 20 µm). **b** Quantification of EDV counts per × 40 fields is presented. **c** Kaplan–Meier analysis showed that S1PR1-positive non-TNBC patients had a poorer prognosis. **d** Survival of S1PR1-positive TNBC patients was not significantly affected. **e** Human breast cancer specimens were analyzed by immunohistochemistry. Positive expression and negative expression of S1PR1 (a, b), VE-cadherin (c, d), β-catenin (e, f) ( × 200, bars 20 µm). *p *<* 0.05

The S1PR1 expression levels in breast cancer samples were analyzed in the context of the detailed clinical and pathological information. The S1PR1 protein was highly expressed in 63 of the 100 breast cancer sample tissues and weakly expressed in the remaining 37 samples (Table 1). S1PR1 expression was correlated with the clinical stage, lymphatic metastasis, and VM (*p* ≤ 0.005, Table 1). The channels that stained Periodic Acid-Schiff (PAS) positive and CD31 negative and that contained red blood cells were considered VM (red arrows, Fig. 1a). Channels that stained positive for both PAS and CD31 were defined as EDV (black arrows, Figs. 1a, b). After detailed comparison of microvessel density between low and high S1PR1 groups, we found that S1PR1 was related to microvessel density (*p* ≤ 0.005, Table 1).

**Table 1:** The differences of postoperative clinical data between S1PR1 group and control group

Variables	S1PR1		*x* ^2^	*p-*Value
	– (%)	+ (%)		
Age				
<50	21	37	0.037	0.847
≥50	16	26		
Tumor size				
<3	14	23	0.018	0.894
≥3	23	40		
Grade				
I/II	28	44	0.394	0.530
III	9	19		
TNM stage				
I/II	35	49	4.905	0.027
III/IV	2	14		
Lymphatic metastasis				
No	26	29	5.533	0.019
Yes	11	34		
Triple negative				
No	20	45	3.093	0.079
Yes	17	18		
VM				
No	26	58	8.237	0.004
Yes	11	5		

*VM* vasculogenic mimicry, *S1PR1* sphingosine-1-phosphate receptor 1

*Statistically significant *p* < 0.05

S1PR1 expression was not significantly different between triple-negative breast cancer and non-triple-negative breast cancer. In previous studies, a high level of VM expression was correlated with triple-negative breast cancer. The Kaplan–Meier survival analysis suggested that in non-triple-negative breast cancer, S1PR1 was correlated with poor patient survival, whereas the correlation was not significant in the triple-negative group (Figs. 1c, d).

Statistical analysis of the immunohistochemical staining showed that VE-cadherin was highly expressed in the S1PR1-negative breast cancer tissues, and VE-cadherin was expressed at a low level in the S1PR1-positive breast cancer tissues (*p* = 0.013; Table 1; Fig. 1e). Meanwhile, β-catenin was highly expressed in the S1PR1-positive breast cancer tissues, and was expressed at a low level in the S1PR1-negative breast cancer tissues (*p* = 0.020; Table 2; Fig. 1e).

**Table 2:** The differences of VE-cadherin and β-catenin between S1PR1 group and control group

Variables	S1PR1		*x* ^2^	*p-*Value
	– (%)	+ (%)		
VE-cadherin				
Negative	10	33	6.113	0.013
Positive	27	30		
β-Catenin				
Negative	27	31	5.405	0.020
Positive	10	32		

*S1PR1* sphingosine-1-phosphate receptor 1

^*^Statistically significant *p* < 0.05

### S1PR1 promoted EDV and prevented VM formation

We examined whether these cellular functions were affected by S1PR1 in the in vitro experiments. Cell lines with high and low S1PR1 expression were selected from several breast cancer cell lines (HS-578T, MDA-MB-231, MCF-7, T-47D, and BT-474) (Fig. S1A). MCF-7 (high S1PR1 expressing cells) were transfected with the shControl RNA or S1PR1 shRNA (shS1PR1). MDA-MB-231 cells, which constitutively express a low level of S1PR1, were transfected with the control vector or S1PR1 (Fig. S1B, C, D). The experimental results were examined by western blotting.

**Fig. 2:**
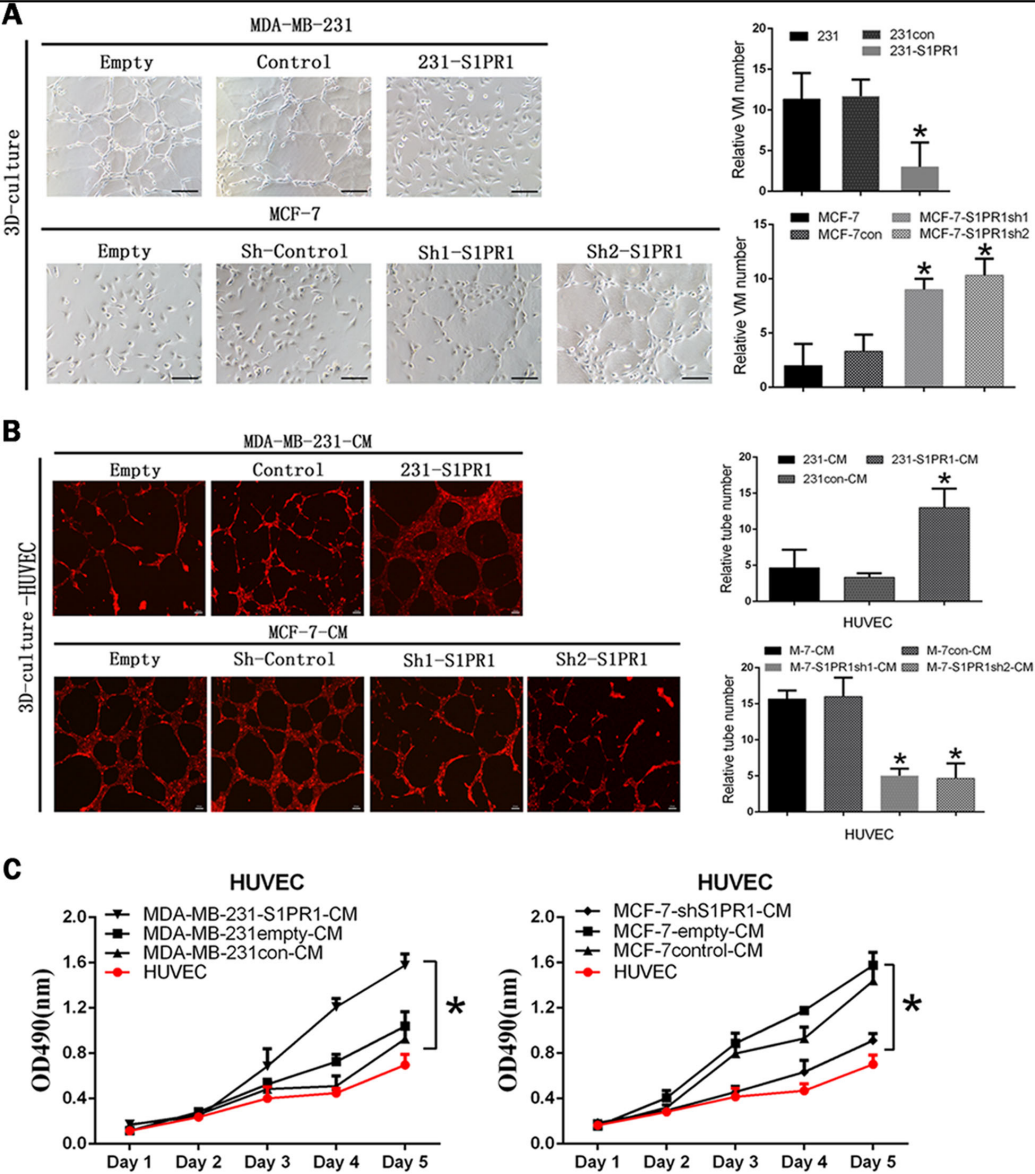
Effect of sphingosine-1-phosphate receptor 1 (S1PR1) on vasculogenic mimicry (VM) or endothelium-dependent vessel (EDV) in human breast cancer cells and the proliferation of human umbilical vein endothelial cells (HUVECs). **a** VM channel formation was observed in the S1PR1-silenced groups; in contrast, the S1PR1-overexpressing groups underwent little channel formation (100 × , bar 50 µm). **b** HUVECs were cocultured with CM from MDA-MB-231 cells (control/S1PR1 upregulated) or MCF-7 cells (shControl/S1PR1 downregulated) (40 × , bar 100 µm). Channel formation was increased in the S1PR1 upregulated groups compared with the control groups. The S1PR1 downregulated groups gave the opposite result. **c** The tumor supernatant from the MDA-MB-231 cells (control/S1PR1 upregulated) or MCF-7 cells (shControl/S1PR1 downregulated) was collected to treat HUVECs, which were analyzed by MTT. HUVEC proliferation was increased in the S1PR1 upregulated groups compared with the control groups. The S1PR1 downregulated groups gave the opposite result. The mean ± SD is shown. *p < 0.05 (*n* = 3)

**Fig. 3:**
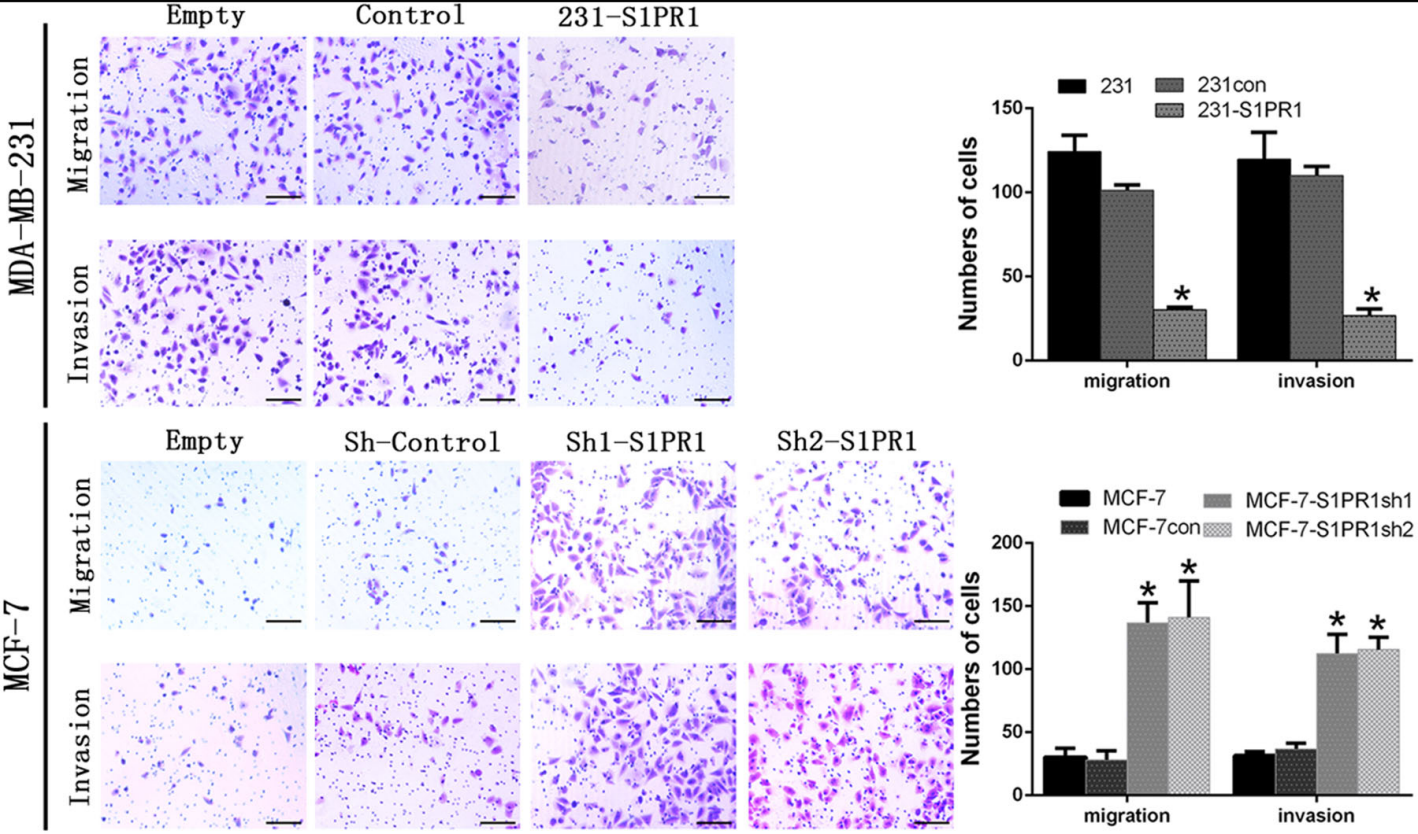
Effects of sphingosine-1-phosphate receptor 1 (S1PR1) on the migration, invasion in human breast cancer cells (100 × , bar 50 µm). Overexpressed S1PR1 reduced the migration and invasion of S1PR1-transfected cells, whereas silenced S1PR1 promoted the migration invasion of S1PR1-transfected cells. Histograms show the numbers of migrated cells. The mean ± SD is shown. *p *<* 0.05 (*n* = 3)

We divided the cell lines with high and low S1PR1 expression and their corresponding downregulated and upregulated groups in the subsequent experiments. To explore the relationship between S1PR1 and VM, we performed tube formation assays in vitro. The results showed S1PR1 expression inhibited 3D channels in MCF-7 cells, whereas S1PR1 deficiency significantly promoted VM formation (Fig. 2a). The MCF-7shS1PR1 group formed more tubes than the cells transfected with the empty plasmid and blank control (Fig. 2a). We performed HUVECs in vitro tube formation assays with CM from the transfected cells. Tube formation by HUVECs was increased after treatment with CM from the MDA-MB-231-S1PR1 cells compared with that of CM from the control cells (Fig. 2b). Conversely, S1PR1 knockdown in MCF-7 cells decreased the HUVEC tube formation more than the shControl (Fig. 2b). These results showed that S1PR1 promoted EDV, whereas S1PR1 deficiency contributed to VM generation. The proliferative capacity of the HUVECs was related to tubule formation. HUVECs were cultured with CM prepared from each group of transfected tumor cells. The results confirmed that the S1PR1-overexpressing groups were more conducive to HUVEC proliferation than the groups with low S1PR1 expression (Fig. 2c). This finding indicated that breast cancer cells might promote the formation of an endothelium-dependent vascular system by stimulating endothelial cell proliferation. Generally, VM formation is related to migration and invasion; therefore, we performed the relevant experiments. The results showed that low S1PR1 expression promoted the migration and invasion of MDA-MB-231 cells. After upregulating S1PR1 in the MDA-MB-231 cells, the migration and invasion abilities were significantly weakened (Fig. 3). We verified this phenomenon by knockdown S1PR1 in MCF-7 cells (Fig. 3). These results suggested that S1PR1 promoted EDV by stimulating HUVEC cell proliferation while inhibiting VM formation in breast cancer cells via weakening their migration and invasion abilities.

### S1PR1 increased β-catenin expression and led to VE-cadherin deficiency

**Fig. 4:**
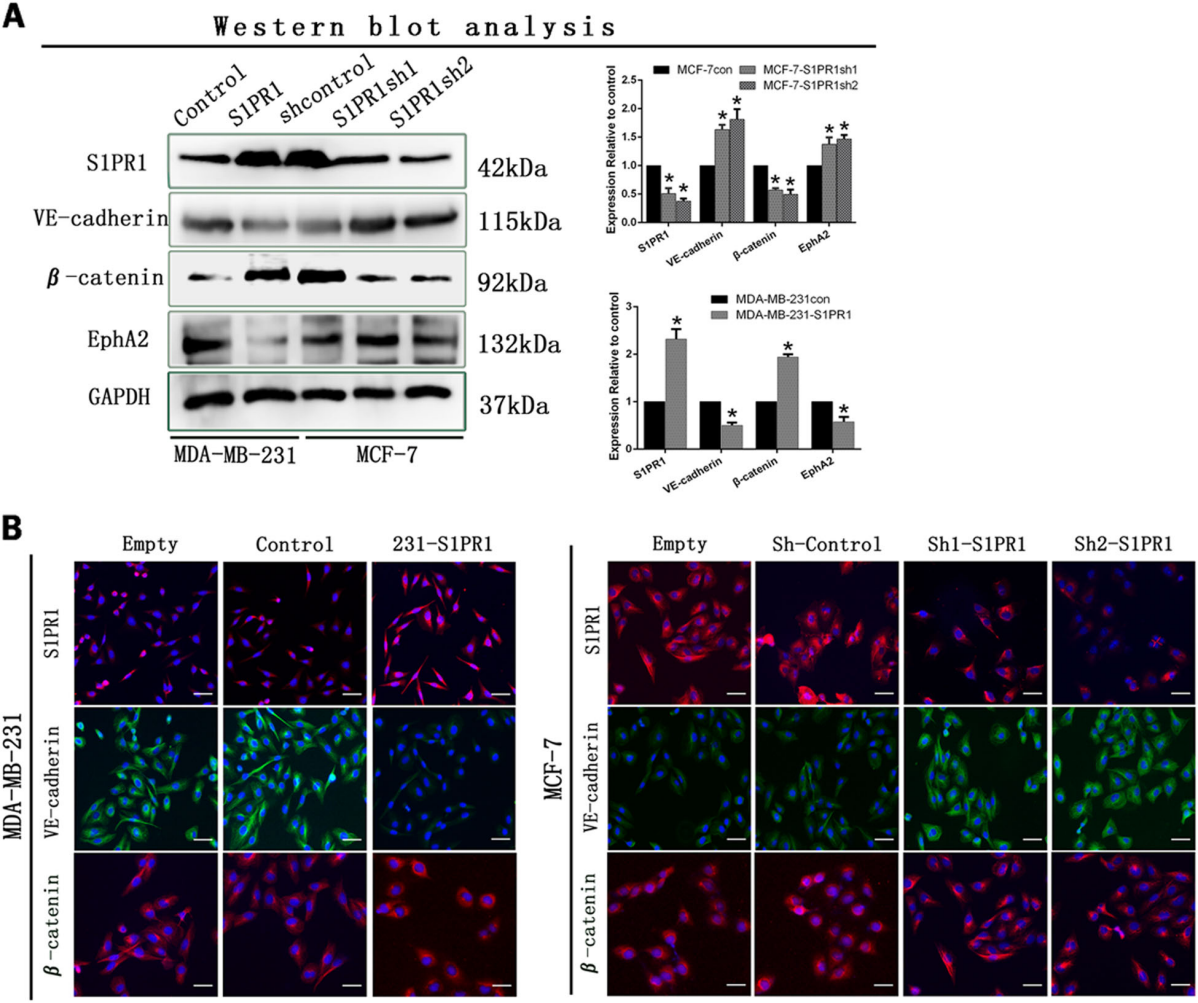
Sphingosine-1-phosphate receptor 1 (S1PR1) suppressed VE-cadherin expression and promoted β-catenin expression. **a** Western blots showed that downregulation of S1PR1 increased the expression levels of VE-cadherin and weakened β-catenin expression. **b** Immunofluorescence staining. S1PR1 may induce a positional change in β-catenin in breast cancer cells and decrease VE-cadherin expression (bar, 50 µm). The mean ± SD is shown. *p *<* 0.05 (*n* = 3)

We used western blot assays to explore molecular mechanisms underlying the ability of S1PR1 to regulate angiogenesis. These data indicated that downregulation of S1PR1 in MCF-7 cells increased the expression of VE-cadherin and EphA2 expression and decreased β-catenin expression (Fig. 4a). Upregulation of S1PR1 in MDA-MB-231 cells increased β-catenin expression and decreased VE-cadherin and EphA2 expression (Fig. 4a). To elucidate the underlying mechanism, we performed immunofluorescence experiments and found that VE-cadherin expression was decreased in cells with high S1PR1 expression and that β-catenin expression shifted from the cell membrane to the cytoplasm and nucleus. VE-cadherin expression was increased, and β-catenin expression was biased toward the cell membrane (Fig. 4b). Therefore, we speculated that S1PR1 promoted angiogenesis by regulating the connection between VE-cadherin and β-catenin.

### S1PR1 promoted the separation of VE-cadherin from β-catenin by increasing VE-cadherin phosphorylation

**Fig. 5:**
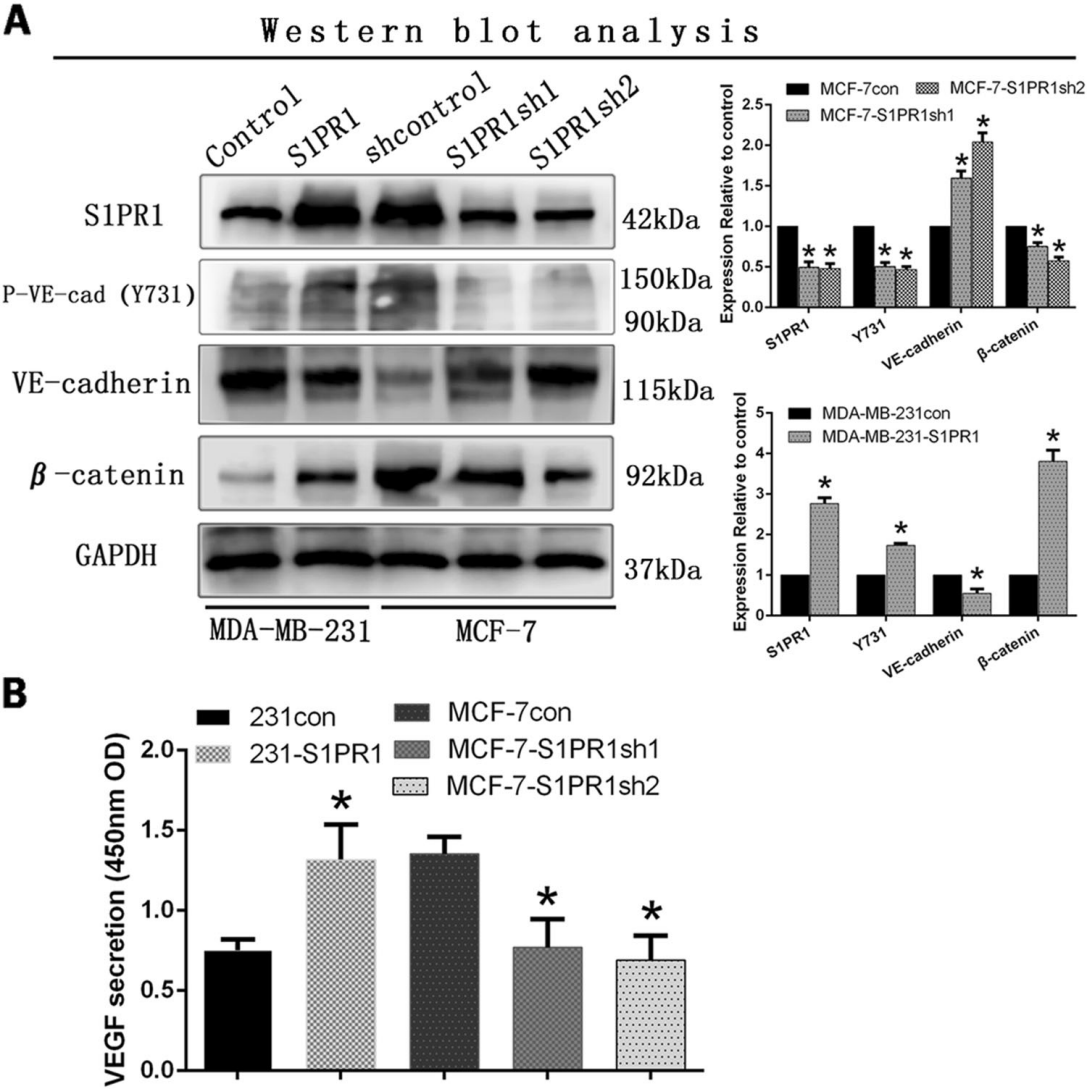
Sphingosine-1-phosphate receptor 1 (S1PR1) promoted VE-cadherin phosphorylation and the secretion of vascular endothelial growth factor (VEGF). **a** Western blot showing that S1PR1 overexpression promoted phosphorylation of the Y731 site of VE-cadherin and β-catenin expression compared with that of the control groups. **b** VEGF protein expression was determined using an ELISA in the cell culture supernatant (OD 450 nm). S1PR1 overexpression increased VEGF secretion compared with that of the control groups. Downregulation of S1PR1 groups suppressed VEGF secretion. The mean ± SD is shown. *p < 0.05 (*n* = 3)

Previous reports have indicated that the junction of VE-cadherin and β-catenin lies in the intracellular Y731 site of VE-cadherin. Therefore, we examined the difference in the Y731 site of VE-cadherin through western blotting. We found that the phospho-VE-cadherin (Y731) levels were increased in the S1PR1-overexpressing MDA-MB-231 cells compared with those in the control and empty cells (Fig. 5a). However, the MCF-7-shS1PR1 cells gave opposite results (Fig. 5a). Therefore, VE-cadherin phosphorylation was the key point that triggered β-catenin to break away from VE-cadherin and was also the key point leading to VE-cadherin internalization and decomposition. We used the tumor cell supernatant to evaluate VEGF expression in MDA-MB-231 cells (overexpressing S1PR1 or control) and MCF-7 cells (transfected with the shControl or shS1PR1) through ELISAs. We found that S1PR1 overexpression in the MDA-MB-231 cells increased VEGF expression compared with that of the control. In contrast, downregulation of S1PR1 in MCF-7 cells significantly suppressed VEGF expression (*p* < 0.001, Fig. 5b). We confirmed that S1PR1 overexpression in breast cancer increased VEGF expression and secretion.

### S1PR1 regulated the phosphorylation of VE-cadherin by activated RhoA

**Fig. 6:**
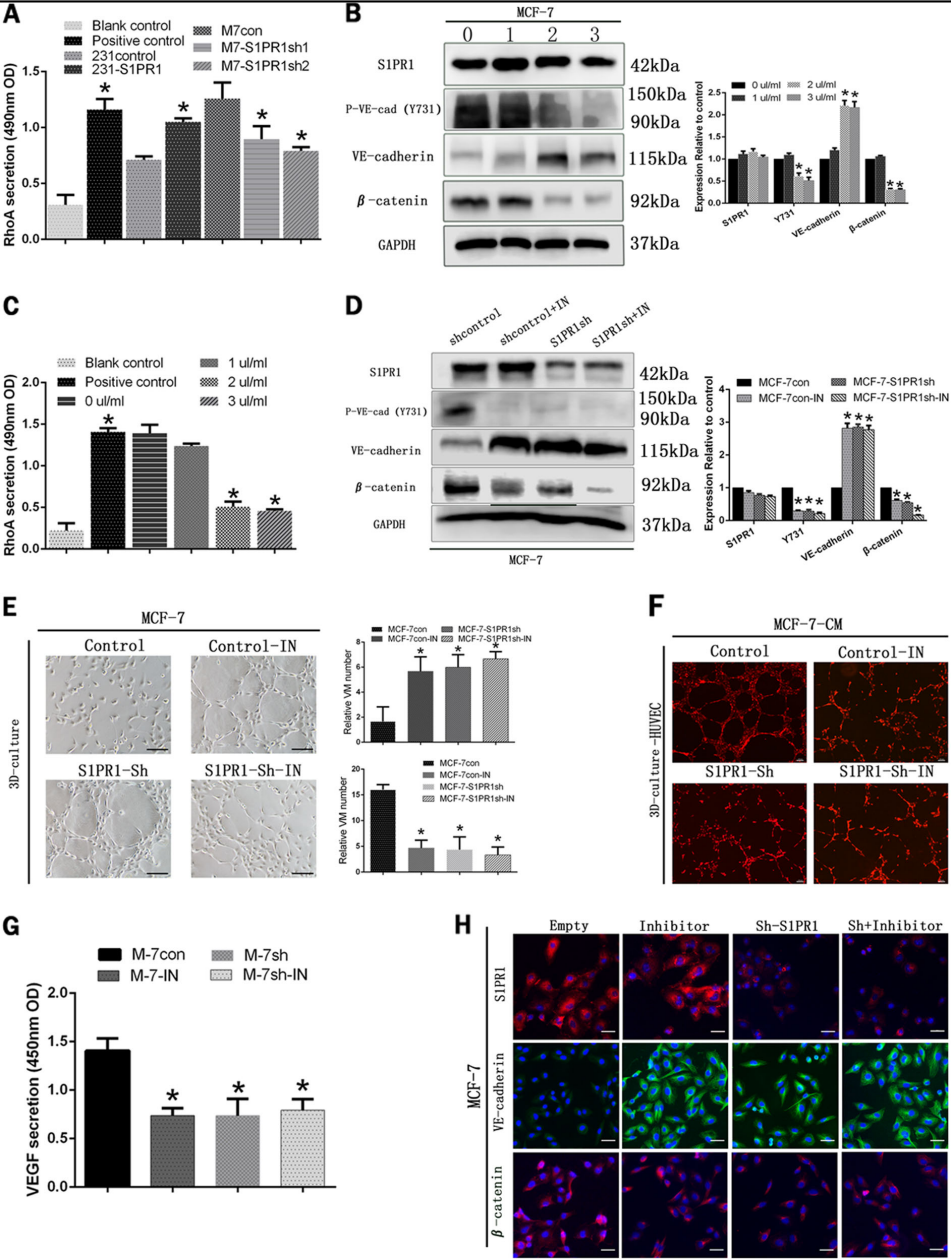
The RhoA-mediated signaling pathway participated in sphingosine-1-phosphate receptor 1 (S1PR1)-mediated vasculogenic mimicry (VM) and endothelium-dependent vessel (EDV). **a** RhoA measurement in the different treatment groups by G-LISA. **b**, **c** The protein levels of S1PR1, VE-cadherin, VE-cadherin (Y731), and β-catenin and RhoA activation in MCF-7 cells after treatment with various RhoA inhibitor concentrations for 24 h. Treatment with 2 µl/ml of the RhoA inhibitor was the optimum concentration. **d** After treatment with 2 µl/ml of the RhoA inhibitor, the S1PR1, VE-cadherin, VE-cadherin (Y731), and β-catenin protein levels in the control or shS1PR1 MCF-7 cells are shown. **e** The RhoA inhibitor restrained vascular endothelial growth factor (VEGF) secretion by MCF-7 cells. **f**, **g** The RhoA inhibitor induced VM channel formation (100 × , bar 50 µm) and inhibited the number of EDVs in MCF-7 cells in 3D culture (40 × , bar 100 µm). **h** After treatment with the RhoA inhibitor, the S1PR1, VE-cadherin and β-catenin protein levels changed, as shown by immunofluorescence staining (100 × , bar 50 µm). The mean ± SD is shown. *p < 0.05 (*n* = 3)

From the previous results, we know that the junction site of VE-cadherin and β-catenin (Tyr731 sites of VE-cadherin) can be phosphorylated by Rho. At the same time, it is interesting that the regulation of S1PR1 in cells depends largely on the activation of RhoA. Thus, a novel G-LISA assay that was very sensitive to activated RhoA was used to detect RhoA activity. The results indicated that cells with high S1PR1 expression (MDA-MB-231-S1PR1 and MCF-7) had higher RhoA activity than cells with low S1PR1 expression (MDA-MB-231 and MCF-7sh) (Fig. 6a). Through further experimentation, we explored the effect of S1PR1 on VE-cadherin phosphorylation by RhoA activation. To confirm that RhoA was indeed necessary for VE-cadherin phosphorylation, we treated the MCF-7 cells with a Rho inhibitor (a cell-permeable compound that directly targeted the Rho GEF binding domain (Kd = 354 nM for RhoA), thereby preventing Rho from interacting with its GEFs). First, we selected the optimal concentration of the inhibitor by western blotting and G-LISA. Then, 2 µl/ml of the RhoA inhibitor (depending on the state of the cells and the effect of RhoA inhibitor) was added to each group, and the cells were stimulated for 24 h before the next experiment (Figs. 6b, c).

After addition of the Rho inhibitor, the S1PR1 expression level did not change significantly, but its downstream factors underwent a series of changes. Phospho-VE-cadherin (Y731) and β-catenin expression was decreased significantly. Conversely, VE-cadherin expression was enhanced (Fig. 6d). To determine whether S1PR1 promoted tumor angiogenesis and decreased VM formation via RhoA signaling, we used a RhoA inhibitor to block the separation of VE-cadherin and β-catenin in the tube formation assays. We found that when we inhibited RhoA activation, human breast cancer cells could form channels more easily (Fig. 6e). However, the number of endothelial cell channels was greatly reduced by treatment with CM from the MCF-7-inhibitor group (Fig. 6f). We speculated that substances secreted by MCF-7 cells were blocked by the Rho inhibitor. Therefore, we evaluated the changes in VEGF by ELISA and found that expression in the inhibitor and S1PR1 downregulated groups was significantly reduced compared with that of the control group (Fig. 6g). The immunofluorescence studies demonstrated that treatment with the inhibitor shifted β-catenin localization from the nucleus to the cell membrane. The results showed that β-catenin could promote VEGF secretion accompanied by part of β-catenin entering the nucleus (Fig. 6h). Collectively, these findings suggested that S1PR1 was a key factor regulating the transformation of the two vascular patterns. S1PR1 promoted tumor endothelial-dependent vessel and decreased VM formation via RhoA signaling.

### S1PR1 signaling could be inhibited by VPC 23019 in vitro

**Fig. 7:**
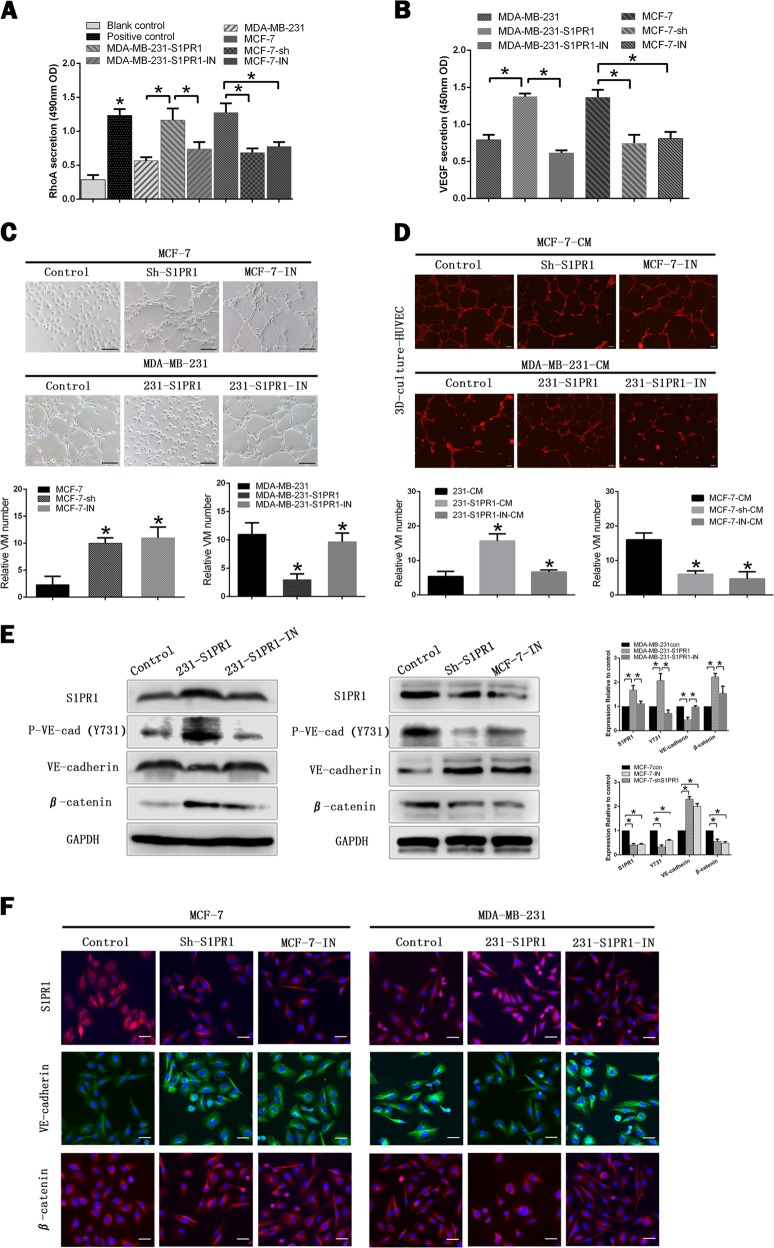
Sphingosine-1-phosphate receptor 1 (S1PR1) signaling could be inhibited by VPC 23019, sphingosine-1-phosphate receptor 1 antagonist, in vitro. **a**, **b** RhoA and vascular endothelial growth factor (VEGF) measurement in the different treatment groups by G-LISA and ELISA. **c**, **d** MCF-7-shS1PR1, MCF-7-IN, and 231-S1PR1-IN groups promoted vasculogenic mimicry (VM) channel formation (100 × , bar 50 µm) and reduced the number of endothelium-dependent vessels (EDVs) in 3D culture (40 × , bar 100 µm). **e** The protein levels of S1PR1, VE-cadherin, VE-cadherin (Y731), β-catenin were changed in MCF-7-shS1PR1, MCF-7-IN, and 231-S1PR1-IN groups. **f** The change of S1PR1, VE-cadherin and β-catenin was shown by immunofluorescence staining (100 × , bar 50 µm). Shown are mean ± SD, *p < 0.05

After adding VPC 23019, S1PR1 antagonist, we evaluated the downstream factors of RhoA and VEGF by G-LISA and ELISA. We found the expression of RhoA and VEGF were significantly reduced in both VPC 23019 groups (MCF-7-IN, MDA-MB-231-S1PR1-IN) and S1PR1 low expression groups (MCF-7-shS1PR1, MDA-MB-231) (Figs. 7a, b). In 3D cultures assay the ability of VM formation was greatly promoted in VPC 23019 groups (MCF-7-IN, MDA-MB-231-S1PR1-IN) and S1PR1 low expression groups (MCF-7-shS1PR1, MDA-MB-231), whereas the number of endothelial cell channels was reduced (Figs. 7c, d). It was further verified by western blotting and immunofluorescence that the results obtained by VPC 23019 groups (MCF-7-IN, MDA-MB-231-S1PR1-IN) were similar with S1PR1 low expression groups (MCF-7-shS1PR1, MDA-MB-231) (Figs. 7e, f).

### S1PR1 acted as a VM suppressor in a xenograft tumor model

**Fig. 8:**
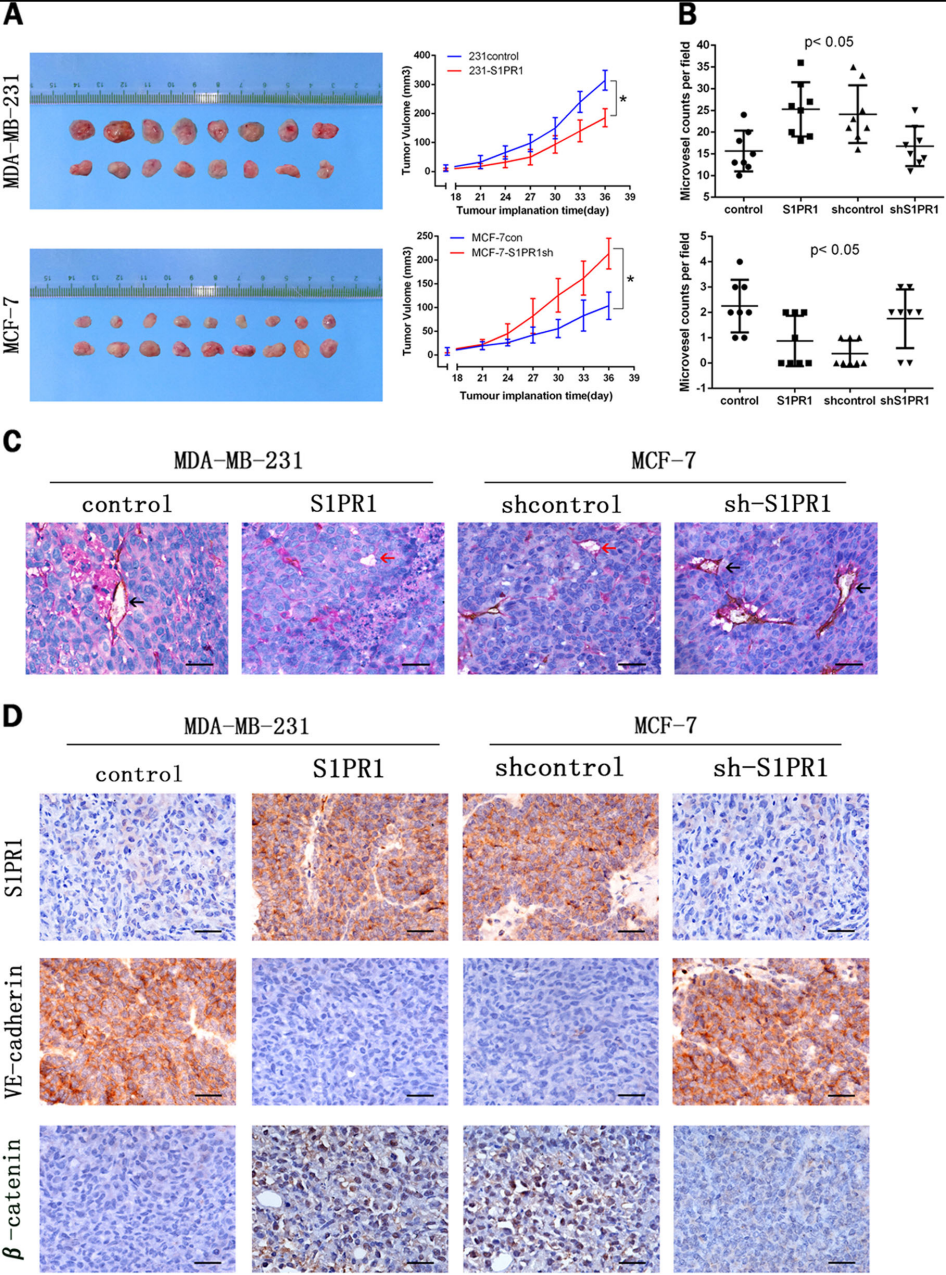
Effects of sphingosine-1-phosphate receptor 1 (S1PR1) on tumor growth and vasculogenic mimicry (VM) in vivo. **a** Tumor growth curves showed that tumor growth in the S1PR1 overexpression groups was slower than that in the control, whereas tumor growth in the S1PR1 downregulation groups was faster than that in the control. **b** The numbers of VM tubules and the number of EVD per 40 × field of view. **c** VM (red arrow) and endothelium-dependent vessels (EDVs; black arrow) channels with or without red blood cells revealed by endomucin/PAS double-staining (400 × , bar 50 µm). The S1PR1, VE-cadherin, and β-catenin expression levels were evaluated by IHC staining (400 × ). *p < 0.05

To further investigate the function of S1PR1 in vivo, stably transfected cells (MCF-7sh-S1PR1 and MDA-MB-231-S1PR1) and their respective controls cells were subcutaneously injected into BALB/c-nu/nu mice. By plotting the tumor growth curves, we found that the tumor sizes began to differ after 18 days of inoculation. Tumors of the S1PR1-overexpressing group grew more slowly than those of the control group (Fig. 8a). In contrast, tumors of the shS1PR1 group grew more quickly than those of the controls. Endomucin/PAS double staining validated the relationship between S1PR1 expression and VM or EDV in vivo. The channels that stained PAS positive and Endomucin negative were considered VM (red arrows, Fig. 7c). Channels that stained positive for both PAS and Endomucin were defined as EDV (black arrows, Fig. 7c). Microscopic examination indicated that the number of VM events was significantly reduced in the S1PR1-overexpressing group compared with that of the control group, whereas the number of EDV was significantly increased in the S1PR1-overexpressing group compared with that of the control group (Figs. 8b, c). Moreover, we evaluated the VE-cadherin and β-catenin expression levels by IHC staining. We found that VE-cadherin expression was significantly lower in the S1PR1-overexpressing group than in the control group. In contrast, VE-cadherin expression was higher in the low S1PR1 expression group than in the control group. However, β-catenin expression was consistent with S1PR1 expression; β-catenin expression was high in the S1PR1-overexpressing group compared with that of the control group (Fig. 8d). Together, these results showed that S1PR1 could prevent the VM formation and promote endothelial-dependent vessel in vivo.

## Discussion

Tumor angiogenesis is a process by which tumors induce the development of new blood vessels to ensure a blood supply in solid tumors^26^. Angiogenesis in tumors includes angiogenic mimicry in addition to EDVs. A variety of receptor-mediated signaling pathways have been found that coordinate different angiogenesis patterns in tumor cells^11,27^. However, the switch mechanisms between EDVs and VM remain unclear. In this study, we found that S1PR1 could induce the switch in human breast cancer.

S1PR1 was originally named EDG1 (the endothelial differentiation gene 1)^28^. S1PR1 has different functions in different cell types. In endothelial cells, S1PR1 was suggested to play roles in the morphogenic differentiation of vascular endothelial cells and in angiogenesis. In adult organisms, S1PR1 prevents a leaky vasculature and promotes tighten junctions^29^. In the immune system, S1PR1 can recruit immune cells to be reintroduced into the lymphatic circulation, especially by guiding activated T cells from nonlymphoid tissues into the lymphatics^30^. S1PR1 regulates different molecules in various types of cancer, of which the most important aspect is associated with angiogenesis^16,21,31^. In our investigation, high S1PR1 expression was found to lead to reduced VE-cadherin expression in cancer cell membranes. This phenomenon was most likely due to destruction of the VE-cadherin–β-catenin complex, resulting in localization of VE-cadherin in the relaxed tumor cell membrane^32^. The VE-cadherin–β-catenin complex is mostly expressed in endothelial cells and plays an important role in regulating vascular permeability^33^. The VE-cadherin–β-catenin complex is also expressed in highly aggressive tumor cells, and its downregulation implies the loss of VM formation^11,32^. Therefore, VE-cadherin expression is a marker associated with VM formation^10^. VE-cadherin is internalized and decomposed to prevent VM formation. In some ways, the degree of tumor aggression may be reduced. However, we found that β-catenin fell off the compound and entered the nucleus, which could improve VEGF transcription in human breast cancers. As a tumor-secreted growth factor, VEGF is the principal driver that stimulates endothelial cell proliferation and migration^34–36^. Therefore, the number of endothelium-dependent microvessels was increased.

S1PR1 is a G-protein-coupled receptor whose interaction with RhoA can regulate many functions of tumor cells, including cell growth, survival, migration, and morphogenesis^17,37^. In our study, S1PR1 was associated with two angiogenic patterns in breast cancer by regulating the RhoA signal pathway. RhoA is the founding member of the Rho GTPase family, which serves as an intracellular molecular switch by cycling between a GTP-bound active form and a GDP-bound inactive form^38–41^. RhoA is an important factor in the regulation of VE-cadherin phosphorylation^37,42^. Activated RhoA can induce VE-cadherin phosphorylation at position Y731 where VE-cadherin binds to β-catenin to make the structure of the VE-cadherin compound unstable. This dynamic is consistent with our experimental results.

Antiangiogenic drugs have been used to suppress the development of all solid tumors. To decrease tumor growth, many studies use Sphk inhibitors to inhibit S1P synthesis in tumor cells. However, the inhibitors have yielded contradictory results^25^, possibly because current antiangiogenesis treatments focus only on classical angiogenesis. However, anti-EDV may increase VM formation leading to tumor metastasis^14^. In our experiments, we found that S1PR1 was the key to regulating the switch between the two angiogenic modes. Thus, in breast cancer with high S1PR1 expression, antiangiogenic drugs should combine anti-EDV with anti-VM.

In the immunohistochemical analysis of human breast cancer tissue, S1PR1 expression was closely related to lymphatic metastasis^43^. Previously, a large body of experimental evidence has demonstrated that S1PR1 may trigger lymphocyte egress from lymphoid tissues^44,45^. However, whether S1PR1 in breast carcinoma can recruit lymphocytes to promote the secretion of angiogenesis-related factors by lymphocytes is unknown. Our experiments will continue to work in this direction.

S1PR1 regulated RhoA activation to accelerate VE-cadherin phosphorylation (Y731), leading to increased EDV and reduced VM in human breast cancer cells. These findings may provide a new direction for antiangiogenic therapies for patients with breast cancer.

## Supplementary information

Figure S2

Figure S1

Supplementary Table S1, Supplementary Figure S1, Supplementary Figure S2
