# Supplementary figures and images for: S1PR1 regulates the switch of two angiogenic modes by VE-cadherin phosphorylation in breast cancer
Source: Cell Death Dis. 2019 Feb 27;10(3):200. doi: 10.1038/s41419-019-1411-x (PMC6393557; doi:10.1038/s41419-019-1411-x)

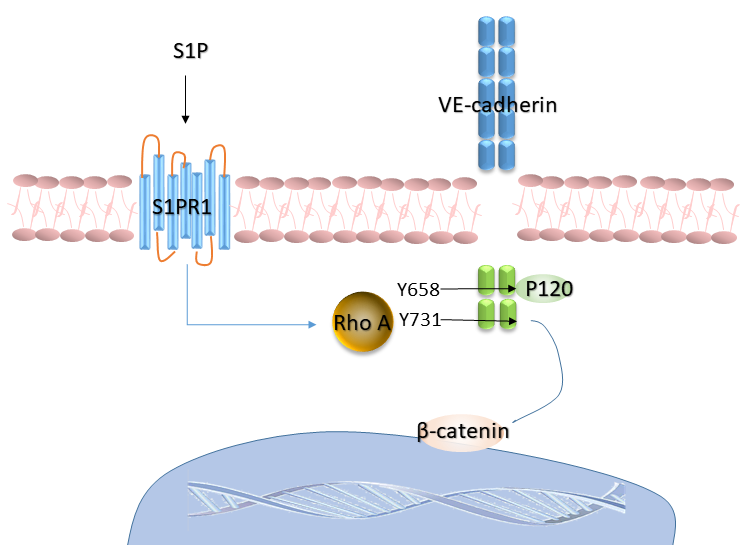

Supplement: Supplementary file 1 — Figure S2 [file 41419_2019_1411_MOESM1_ESM.tif]

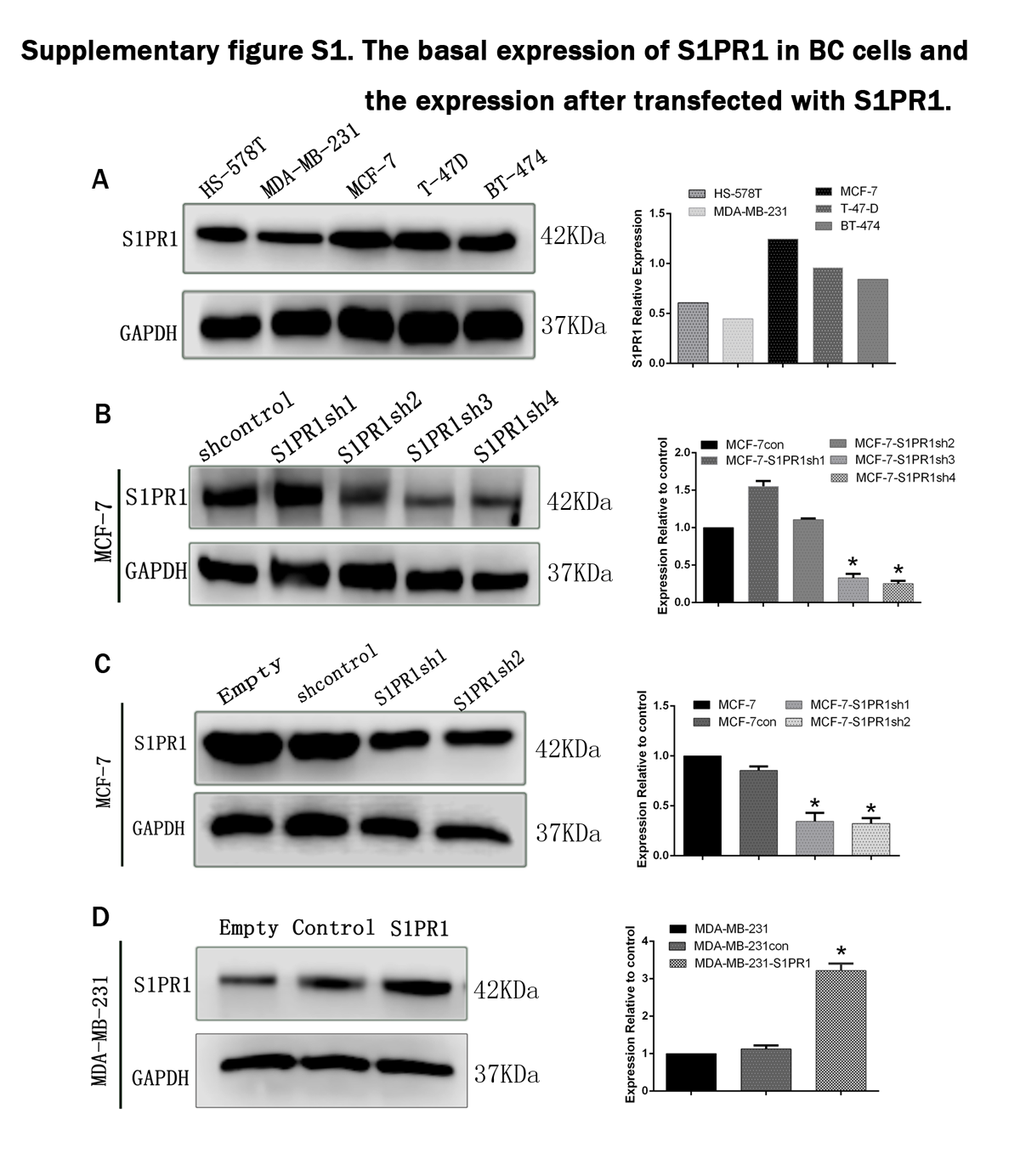

Supplement: Supplementary file 2 — Figure S1 [file 41419_2019_1411_MOESM2_ESM.tif]
