# Supplementary material for: S1PR1 regulates the switch of two angiogenic modes by VE-cadherin phosphorylation in breast cancer
Source: Cell Death Dis. 2019 Feb 27;10(3):200. doi: 10.1038/s41419-019-1411-x (PMC6393557; doi:10.1038/s41419-019-1411-x)
Supplement: Supplementary file 3 — Supplementary Table S1, Supplementary Figure S1, Supplementary Figure S2 [file 41419_2019_1411_MOESM3_ESM.docx]

**Supplementary Table S1**: Antibodies used in this study

| Antibody | Source | IHC Concentration/ WB | Product Number | Manufacture |
| --- | --- | --- | --- | --- |
| S1PR1 | Rabbit | 1:1000(WB) 1:200(IHC) | ab11424 | Abcam |
| VE-cadherin(Y731) | Rabbit | 1:500(WB) | MBS9382362 | MyBioSource |
| VE-cadherin | Rabbit | 1:1000(WB) 1:400(IHC) | ab33168 | Abcam |
| β-Catenin | Rabbit | 1:5000(WB) 1:400(IHC) | ab32572 | Abcam |
| EphA2 | Rabbit | 1:500(WB) | sc-924 | Santa Cruz |
| GAPDH | Mouse | 1:2000(WB) | sc-47724 | Santa Cruz |
| CD31 | Mouse | 1:100(IHC) | ZM-0044 | ZSGB-BIO |
| Endomucin | Rat | 1:800(IHC) | 14-5851-81 | Ebioscience |

Note: IHC: Immunohistochemistry, WB: Western blot

**Supplementary Figure S1** The basic expression of S1PR1 in BC cells and the expression after transfected with S1PR1. (A) The basic expression of S1PR1 in breast cancer cell lines (HS-578T, MDA-MB-231, MCF-7, T-47D and BT-474). (B) MCF-7 was stably transfected with 4 shRNAs and evaluated by Western blotting. (C) The S1PR1sh3 and S1PR1sh4 plasmids were selected to be downregulated as two downregulation groups, hereinafter referred to as S1PR1sh1 and S1PR1sh2. (D) MDA-MB-231 cells were stably transfected with S1PR1 cDNA and control vector. The results were evaluated by Western blotting. Shown are mean ± SD,**p* <0.05

**Supplementary Figure S2** The schematic of S1PR1 on the regulation of endothelium-dependent vessel in breast cancer. S1PR1 (a G-protein-coupled receptor for S1P) binds to RhoA which leads to phosphorylation of VE-cadherin (Y731). β-catenin breaks away from VE-cadherin when VE-cadherin (Y731) was phosphorylated and regulates target genes.
